# Identification of Vital Hub Genes and Potential Molecular Pathways of Dermatomyositis by Bioinformatics Analysis

**DOI:** 10.1155/2021/9991726

**Published:** 2021-09-18

**Authors:** Xueren Ouyang, Yuning Zeng, Xiaotao Jiang, Hua Xu, Yile Ning

**Affiliations:** ^1^Department of Pediatrics, The First Affiliated Hospital of Guangzhou University of Chinese Medicine, Guangzhou, Guangdong Province, China; ^2^The First Clinical School, Guangzhou University of Chinese Medicine, Guangzhou, Guangdong Province, China; ^3^Department of Critical Care Medicine, The First Affiliated Hospital of Guangzhou University of Chinese Medicine, Guangzhou, Guangdong Province, China

## Abstract

Dermatomyositis is an autoimmune disease characterized by severe symmetrical muscle dysfunction and pain. This study was aimed at discovering vital hub genes and potential molecular pathways of DM through bioinformatics analysis, which contributes to identifying potential diagnostic or therapeutic biomarkers and targets. In this study, a total of 915 DEGs in DM samples including 167 upregulated genes and 748 downregulated genes were screened out by the limma package based on the GSE142807 dataset from the Gene Expression Omnibus (GEO) database. Furthermore, the results of Gene Ontology (GO) and the Kyoto Encyclopedia of Genes and Genomes (KEGG) pathway enrichment analysis indicated that these downregulated genes were highly associated with the immune-related biological processes and pathways. Therefore, 41 genes closely related to DM were extracted for further study based on the subcluster analysis through the Molecular Complex Detection (MCODE) software plugin in Cytoscape. Ultimately, 10 hub genes (including ISG15, DDX58, IFIT3, CXCL10, and STAT1) were identified as the potential candidate biomarkers and targets. Besides, we found that the identified hub genes directly or indirectly communicated with each other via molecular signaling pathways on the protein and transcription level. In general, under the guidance of bioinformatics analysis, 10 vital hub genes and molecular mechanisms in DM were identified and the expression of proinflammatory factors and interferon family proteins and genes showed high association with DM, which might help provide a theoretical foundation for the development of point-to-point targeted therapy in the future treatment of DM.

## 1. Introduction

Dermatomyositis (DM) is an autoimmune disease characterized by a skin lesion and myalgia or progressive muscle weakness that results in severe skin rashes, long-term symmetrical muscle dysfunction, and pain, and it causes damage to the function of multiple organs [1]. As a kind of idiopathic inflammatory myopathies (IIM), DM affects both children and adults. In patients with DM, the quality of life is seriously affected. Previous clinical studies on the treatment guidelines of DM mainly emphasized its early diagnosis, treatment, and management. With the deepening of the disease research and clinical literature reports in recent years, the glucocorticoid and immunosuppressant in combination have been recommended for the treatment of the DM, resulting in the reduced incidence of adverse reaction among affected patients [2].

Based on the current research reports, the pathogenesis of DM is mainly closely related to immune disorders and genetic factors [3]. Cytokines, including interleukins and interferon (IFN), are small protein molecules with cell signal transduction functions involved in the development progression of DM [4]. DM is an autoimmune disease mediated by humoral immunity. It is chiefly characterized by infiltration of lymphatic B cells and CD4+ T cells, which are mostly concentrated around blood vessels [3]. Meanwhile, microRNAs (miRNAs) are fundamentally important to the pathogenesis of DM through extensive and precise regulation of various biological processes at the transcriptional level. Recent studies have confirmed that the plasma levels of hsa-miR-4442 in patients with DM changed significantly before and after treatment, which may serve as a potential biomarker [5]. Apart from this, some researchers have also proposed that miR-193b-3p, miR-199b-5p, and miR-665 are related to DM [6]. As an important part of the immune system, Janus Kinase (JAK) family genes are involved in and mediate innate and adaptive immune processes in the human body. The mRNA and protein factors produced by the JAK family genes (JAK1, JAK2, JAK3, and TYK2) are closely related to the regulation of cytokines such as interferon-*γ* (IFN-*γ*), tumor necrosis factor-*α* (TNF-*α*), and interleukin 5 (IL-5) and some main biological processes such as cell proliferation, differentiation, and apoptosis. In recent years, with the deepening of research on the JAK family, it is becoming potential therapeutic management for DM [7, 8]. Moreover, both in vitro pathological tissue experiments and PCR experiments on DM have proven that the mRNA expression level of IFN-related genes (IFITM2, IFIT6, LY6E, etc.) in skeletal muscle tissues of DM patients is more significant. It can promote the secretion of inflammatory factors in muscle tissues and mediate the abnormal apoptosis of skeletal muscle tissues. Other candidate genes include STAT1, DDX58, MX2, and PLSCR1 [9]. In addition, studies have found that mutations in HLA genes can be found in patients with DM, and related genes such as HLA-DRB1, HLA-DQA1, and HLA-DQB1 are associated with susceptibility to DM. It also has indicated that the genetic variation of the HLA gene family and HLA alleles and nonalleles may be related to the pathogenesis of DM [10]. Considering that the main genes involved in DM's pathogenesis and molecular pathways are still unclear, this study was aimed at identifying the key differential genes and molecular pathways that may cause the pathogenesis of DM, ascertaining the biological processes mediated by genes and signaling pathways. On this basis, this study will further investigate the relationship between IFN-related genes and DM's physiology and pathology, providing a theoretical basis for clarifying a new potential biomarker, which may help biomedical researchers and experimental biologists to better understand the pivotal differentially expressed genes and signaling pathways of DM. Furthermore, a bioinformation platform was built to help manage and develop potential therapeutic targets, which will help provide a theoretical foundation for the development of point-to-point targeted therapy in the future treatment of DM [11–13].

Under the guidance of the bioinformatics and network analysis of gene microarrays, it is an effective way to explore gene expression profiles in the pathogenesis of the disease. The gene expression profile and clinical information of the DM cohort were downloaded from the Gene Expression Omnibus database. In addition, to identify hub genes and molecular pathways involved in DM, differentially expressed genes (DEGs) were compared in skin biopsy samples obtained from patients and healthy controls, and these genes were comprehensively assessed through computational biology. The Gene Ontology (GO) and Kyoto Encyclopedia of Genes and Genomes (KEGG) pathway enrichment analysis were applied to construct the protein-protein interaction (PPI) network. What is more, in this study, an analysis was performed using the Molecular Complex Detection (MCODE) software [14].

## 2. Materials and Methods

### 2.1. Gene Expression Profile Data

The expression profile GSE142807 used in this study was retrieved from the NCBI GEO database (http://www.ncbi.nlm.nih.gov/geo). After background correction, the dataset was included with 43 dermatomyositis samples and 5 healthy samples. Patients were selected and sampled according to the clinical diagnostic and histological criteria for DM. The skin biopsies were taken from the patients with or without DM. The research was performed based on the Affymetrix Human Gene 2.1 ST Array (GPL17692, HuGene-2_1-st).

### 2.2. Identification of DEGs

The DEGs between dermatomyositis samples and healthy samples were filtered out by running the limma package in R (version 3.5.3) [15]. According to the previous studies, the genes with a cutoff criterion that ∣logFC | ≥2 (log2 fold change) and *P* value < 0.05 were selected and defined as differentially expressed genes [16–18]. Ultimately, based on the statistical analysis for each dataset, 915 DEGs were filtered out, including 167 upregulated genes and 748 downregulated genes.

### 2.3. GO and KEGG Enrichment Analysis of DEGs

GO knowledgebase, including molecular function (MF), biological process (BP), and cellular component (CC), is the primary bioinformatics tool for linking genomic information to biological annotations, which helps the researchers in the biomedical field to study and analyze the function of genomic data [19]. KEGG is an extensive database composed of sufficient high-throughput genome sequencing data for researchers to understand the roles of somatic cells, organisms, and ecological systems at the level of molecular structure information [20]. To ensure the functions of the DEGs, it is necessary to perform an enrichment analysis on the GO term and KEGG pathways. In the current study, the enrichment results of BP deserve to be emphasized, which helps to interpret the pathophysiological process of dermatomyositis. KEGG enrichment analysis determined the potential molecular signal transduction pathways of DEGs. GO and KEGG enrichment analysis of DEGs was conducted by operating the clusterProfiler [21]. *P* value < 0.05 and gene counts ≥ 10 were considered statistically significant.

### 2.4. PPI Network Construction and Subcluster Analysis

The Search Tool for the Retrieval of Interacting Genes (STRING) database (version 10.0, http://string-db.org/) was applied to predict potential interactions between DEGs and construct the PPI network of DEGs. The PPI network was visualized with an interaction score > 0.4 as a minimum cutoff criterion by running the ggnet-work package [22]. Furthermore, the subclusters in the PPI network of dermatomyositis were established based on the MCODE plugin in Cytoscape software (version 3.7.1) [14, 23]. More options were set at degree cutoff = 2, node score cutoff = 0.2, and *K* − core = 2.

### 2.5. Hub Gene Interrelation Analysis

GeneMANIA (http://genemania.org) is a database for gene function prediction, gene interaction analysis, and gene prioritization. It has been widely recognized and performed by bioinformatics researchers in recent years [24, 25]. Therefore, we extracted and listed the identified hub genes that may be most closely associated with DM from the results of the above subcluster analysis steps and entered them into the GeneMANIA online database to obtain their interrelation analysis. GeneMANIA was performed to visualize the relationships in interactive networks between listed genes and provide other genes associated with the listed one to better clarify how genes directly or indirectly interactively communicate with each other.

## 3. Results

### 3.1. Identification of DEGs

**Figure 1:**
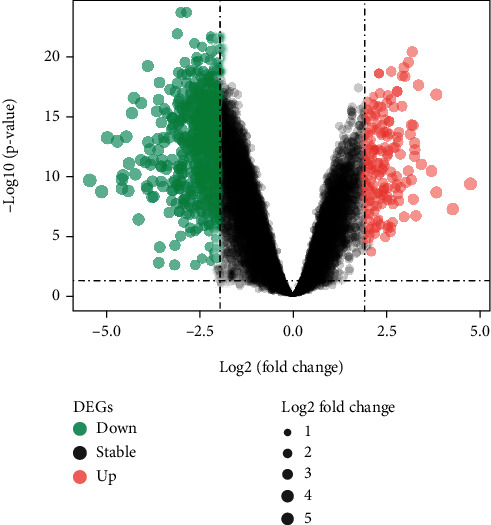
Volcano plot of the differentially expressed genes (DEGs) in dermatomyositis (DM) for dataset GSE142807. Upregulated genes and downregulated genes are shown in red and green, respectively.

**Figure 2:**
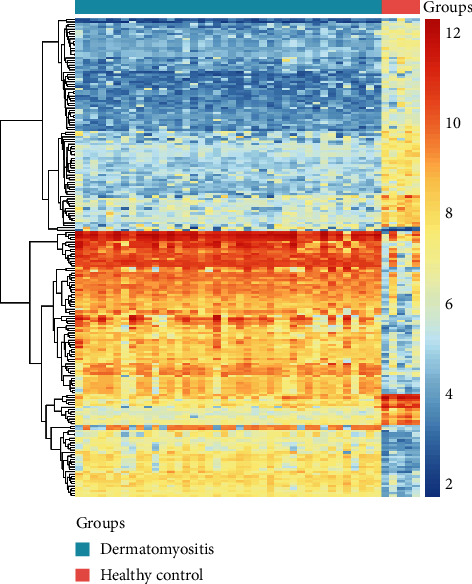
Heatmap of the top 100 upregulated and downregulated genes of the expression dataset for dermatomyositis (DM) by the limma package in R.

After analyzing the discovery datasets (GSE142807) and integrating the analysis of 43 samples from dermatomyositis and 5 healthy samples, we obtained DEGs in DM samples. A total of 167 upregulated genes and 748 downregulated genes in the DEGs that meet the criteria of *P* value and logFC were singled out (Figure 1). The representative top 100 DEGs were visualized in the form of heatmap (Figure 2). The upregulated DEGs and downregulated DEGs are listed in Supplementary Materials (Supplementary Table 1).

### 3.2. GO and KEGG Enrichment Analysis of DEGs

**Figure 3:**
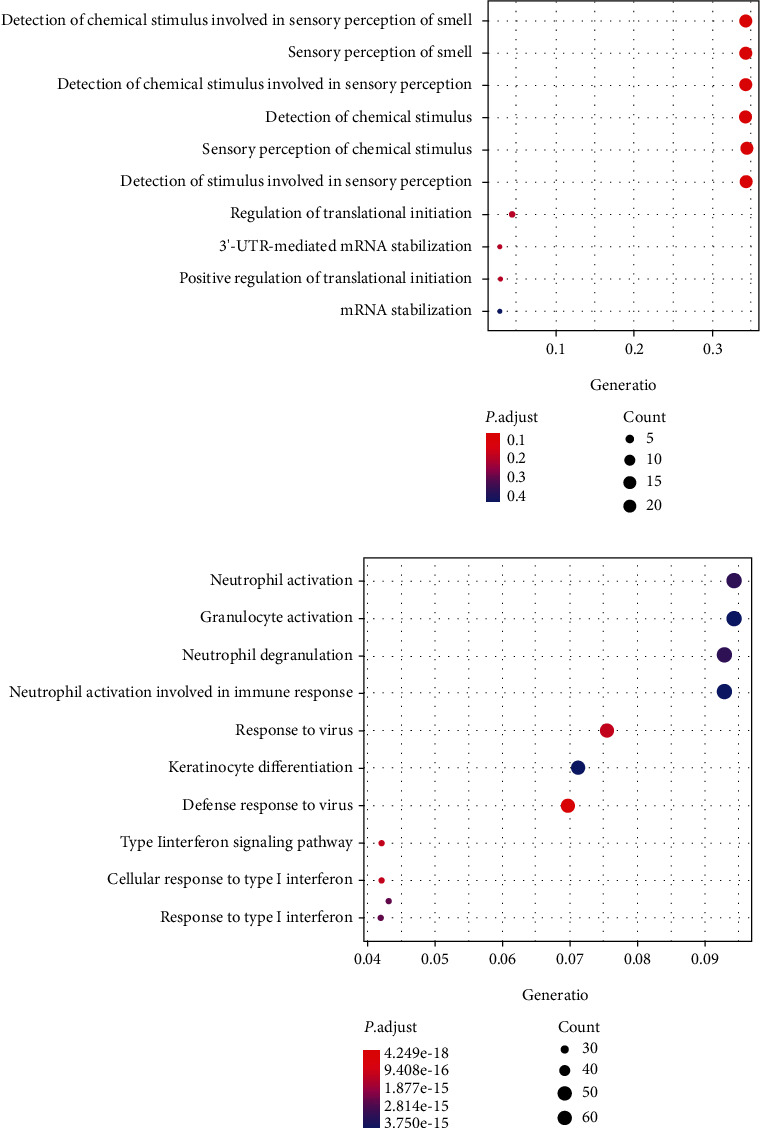
(a) The biological process (BP) of GO enrichment analysis of upregulated genes in dermatomyositis (DM) by the clusterProfiler package in R. (b) The biological process (BP) of GO enrichment analysis of downregulated genes in dermatomyositis (DM) by the clusterProfiler package in R.

The BP of GO enrichment analysis revealed that upregulated DEGs were mainly enriched in the sensory perception of smell, regulation of translational initiation, 3′-UTR-mediated mRNA stabilization, positive regulation of translational initiation mRNA stabilization, and so forth (Figure 3(a)). However, the functional enrichment terms of downregulated DEGs were significantly enriched in the neutrophil activation, granulocyte activation, neutrophil degranulation, neutrophil activation involvement of immune response, response to virus, and so on (Figure 3(b)). And the representative top five GO analysis terms enriched by upregulated and downregulated genes, respectively, for DM are listed in Table 1. In addition, KEGG enrichment analysis revealed that upregulated DEGs were mainly enriched in olfactory transduction and influenza A, while downregulated DEGs were significantly enriched in four KEGG pathways including the endocytosis, Parkinson disease, Kaposi sarcoma-associated herpesvirus infection, and Epstein-Barr virus infection (Table 2).

### 3.3. PPI Network Construction and Subcluster Analysis

**Figure 4:**
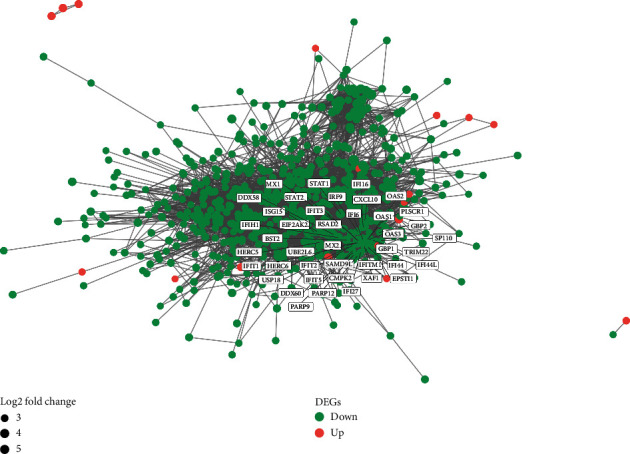
The protein-protein interaction (PPI) network for the DEGs in dermatomyositis (DM) by the ggnet-work package. Genes of the top two subclusters are marked separately.

**Figure 5:**
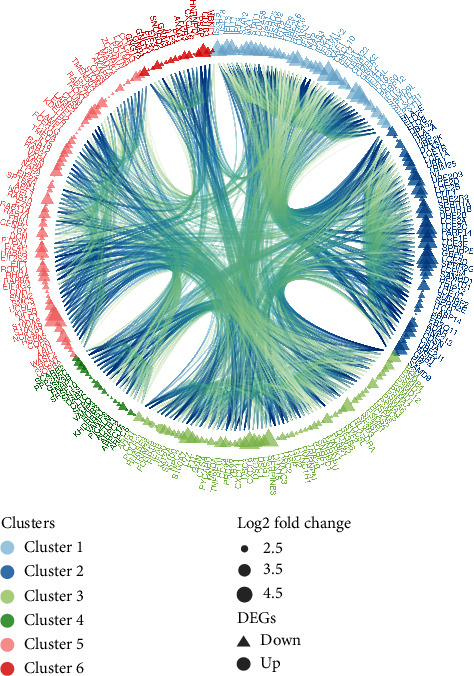
Subcluster analysis for dermatomyositis (DM) by the MCODE Cytoscape software plugin. Genes of the top two subclusters show enhanced connectivity.

**Figure 6:**
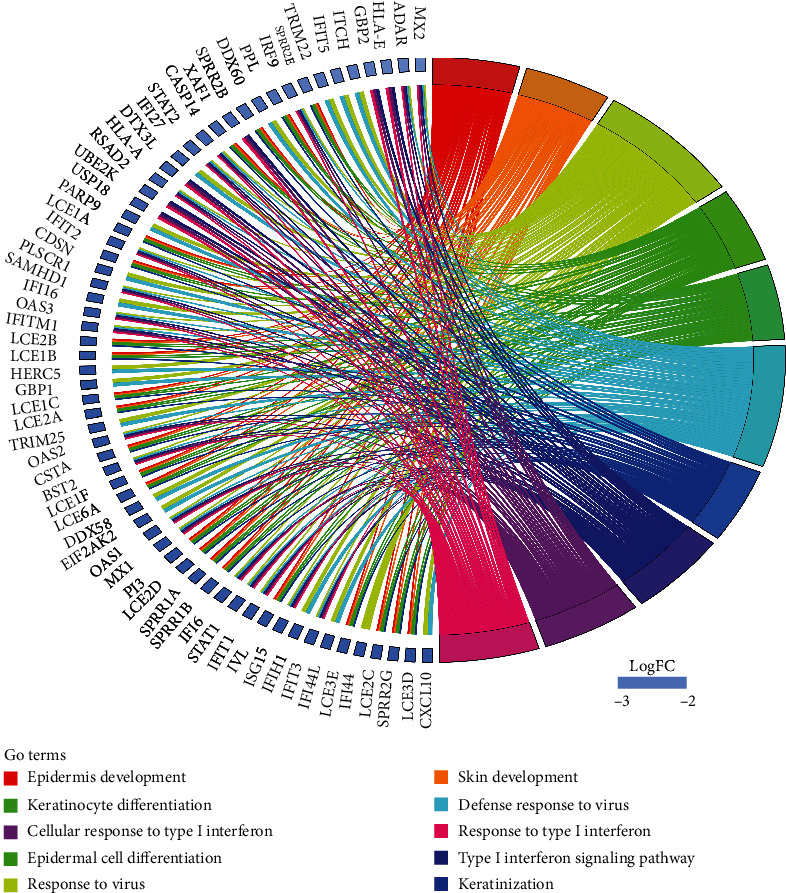
The DM-related genes of the top two subclusters contained in the top ten terms of Gene Ontology (GO) enrichment analysis of biological processes in dermatomyositis (DM).

In sum, 6473 interaction pairs of the DEGs were identified and selected out through the STRING database. On the foundation of these pairs above, the PPI network with 666 nodes of the DEGs that meet the interaction scores greater than 0.4 was visualized (Figure 4). The subcluster analysis for the PPI networks was constructed by running the MCODE software plugin in Cytoscape. Finally, 6 subclusters were visualized and constructed (Figure 5). The subclusters with the MCODE score > 5 were defined as DM-related genes. Hence, a total of 41 correlated genes were selected out from 3 subclusters. The genes of the top 2 subclusters contained in the top 10 GO enrichment analysis terms were systematically processed by the GOplot package (Figure 6), which shows a high correlation with dermatomyositis. Based on the above results and literature review, our team identified 10 genes from the top 2 subclusters that are most closely associated with DM and defined them as vital hub genes. These vital hub genes included C-X-C motif chemokine 10 (CXCL10), retinoic acid-inducible gene 1 (DDX58), interferon-stimulating gene 15 (ISG15), signal transducer and activator of transcription 1 (STAT1), interferon, alpha-inducible protein 6 (IFI6), major histocompatibility complex (HLA-A), tripartite motif-containing 25 (TRIM25), guanylate-binding protein1 (GBP1), etc. Almost most of the downregulated genes identified and selected were associated with the neutrophil activation.

### 3.4. Hub Gene Interrelation Analysis

**Figure 7:**
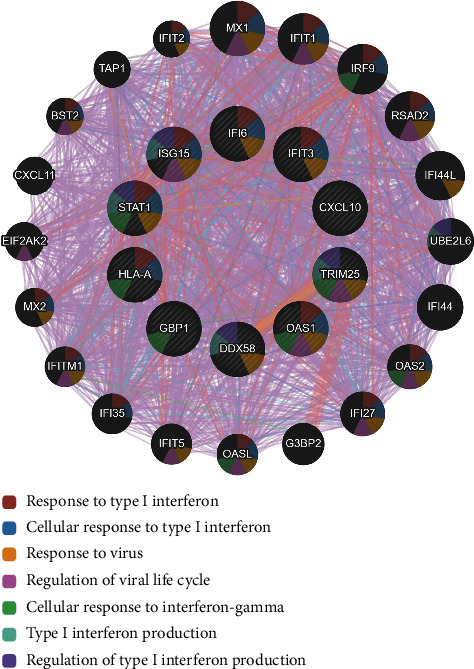
The interrelation analysis of the 10 vital hub genes highly associated with dermatomyositis (DM) by the GeneMANIA. The interactive relationship network between hub genes was mapped and visualized by the GeneMANIA. The different colors of gene nodes represent the enrichment of different gene biological functions.

Based on the above analysis results, we found that the vast majority of hub genes were attributed to the downregulated DEGs and the downregulated DEGs were highly correlated with the immune response. Therefore, we performed the interrelation analysis by assessing the biological process of 10 vital hub genes in GeneMANIA to elucidate their interactions. From the analysis results (Figure 7), it was revealed that there were altogether 2408 links and 20 related genes between 10 vital hub genes, and different colors of gene nodes represent different gene functions. The lines in violet between genes represent coexpression, lines in red represent physical interaction, lines in blue represent colocalization, and the lines in cyan represent a common pathway. As depicted in Figure 7, consistent with the above enrichment results, we observed an enrichment of hub gene differential expression largely in response to type I interferon, cellular response to type I interferon, response to virus, regulation of viral genome replication, regulation of the viral life cycle, cellular response to interferon-gamma, type I interferon production, and regulation of type I interferon production. It suggests that gene function is significantly enriched in the immune system response, which further indicates that multiple genes jointly participate in and mediate the regulation of immune response.

## 4. Discussion

DM is a clinically heterogeneous disease characterized by a skin lesion and myalgia or progressive muscle weakness that results in severe skin rashes [26]. DM has a high clinical incidence, and its complications may be life-threatening. It has already become a significant public health problem. At present, due to the early recognition of DM and the development of medical technology, the diagnosis and treatment of DM have been greatly improved [27].

Nevertheless, the exact pathogenesis of DM has not been clearly described. In the present study, data mining and bioinformatics analysis were performed to identify the DEGs from muscle biopsy samples from patients with DM and healthy controls. This study approach identified the possible hub genes that were highly correlated with the PPI network to pinpoint the potential target genes involved in the pathogenesis of DM [28].

DM-related genes of the top two subclusters were identified using the MCODE analysis and showed a high degree of connectivity in the PPI network. Hub genes of the top two subclusters identified for DM as potential biomarkers are shown in Table 3. These vital hub genes that may be most closely associated with DM included C-X-C motif chemokine ligand 10 (CXCL10), DExD/H-box helicase 58 (DDX58), interferon-stimulating gene 15 (ISG15), major histocompatibility complex, class I, A (HLA-A), interferon induced protein with tetratricopeptide repeats 3 (IFIT3), signal transducer and activator of transcription 1 (STAT1), 2′-5′-oligoadenylate synthetase 1 (OAS1), interferon alpha inducible protein 6 (IFI6), tripartite motif containing 25 (TRIM25), and guanylate-binding protein 1 (GBP1).

CXCL10 encodes a chemokine of the CXC subfamily and ligand for the receptor, which is an important inflammatory factor involved in the immunopathological damage of DM. Studies have shown that the level of CXCL10 in the serum of patients with DM is significantly higher than that of healthy people, which indicates that CXCL10 has significant significance in the study of DM [29]. Furthermore, related studies have shown that CXCL10 can significantly chemoattract inflammatory cells to the lesion. The mechanism is that the IFN-*γ* strongly induced the production of the chemokine CXCL10, which activated the G protein-coupled pathway to participate in Th1-type response by binding to the CXCR3 receptor and mediated T cell migration to the inflammatory site. It is positively correlated with the severity of DM injury [30, 31]. Previous studies showed that CXCL10 was required for the classic PI3K/Akt signaling pathways of inflammatory response, which supported that CXCL10 was involved in regulating immune system response [32, 33].

DDX58, also known as retinoic acid-inducible gene 1 (RIG-I), is retinoic acid- (retinoic acid) induced gene protein I. Previous studies have shown that DDX58 can promote cell secretion of type I interferon and participate in the regulation of immunity [34]. In addition, previous studies have indicated that DDX58 was involved in viral double-stranded (ds) RNA recognition and RIG-I-mediated innate immunity triggered by viruses or injury signals plays an important role in the pathogenesis of DM [35]. Further, related studies have confirmed the overexpression of RIG-I in pathological muscle fibers in DM [34]. Therefore, the RIG-I-mediated regulation of innate immunity might still have a close association with IFN-*β*-dependent mechanisms in DM.

ISG15 is an IFN-*α*/*β*-inducible, intracellular ubiquitin-like protein. Related studies have shown that the level of ISG15 is negatively correlated with the severity of muscle histological damage and positively correlated with exercise capacity (CMAS, MMT) [36, 37]. In addition, for the diagnosis of juvenile DM, the expression of muscle ISG15 is closely related to juvenile DM and may be a potential biomarker for the diagnosis of juvenile DM [37]. In recent years, the description of ISG15 as a central participant in host antiviral response has been widely accepted, and ISG15 could protect and limit the damage to human tissues caused by the viral response through the participation and regulation of the type I interferon signaling pathway. Additionally, the production of IFN-*γ* has a close association with ISG15, which suggests that ISG15 may coregulate with other genes (JAK1, RIG-I, STAT1, etc.) involved in IFN signaling pathways to modulate the response to interferon in DM [38]. Hence, we believe that ISG15 might be a much noteworthy gene in the pathogenesis of DM.

IFIT3, a protein-coding gene, is one of the representative genes of the type 1 interferon system and is involved in the immune response of DM. Besides, it has been found that IFIT3 is an essential part of many biological functions, such as antiviral replication, anticellular abnormal proliferation, and migration. In addition, IFIT3 was involved in the interferon-gamma signaling pathway to modulate response to interferon in the immune system [39]. Recent evidence suggests that IFIT3 is overexpressed in pathological muscle tissues in immunoomic experiments and type I interferon-mediated innate immunity plays a key role in DM [40].

STAT1, a member of the STAT protein family, is involved in gene regulation induced by type I, II, and III interferons as a transcription factor and various basic biological processes [38, 41]. It is worth noting that it is also relevant to immune response. STAT1 encodes proteins that can be activated by diverse ligands, including IFN-*α*, IFN-*γ*, and IL-6, which mediate the expression of various genes. In addition, STAT1 involved in the JAK-STAT signaling pathway regulates the immune system response to interferon, which prompts a high correlation with the interferon system [42]. Moreover, type I interferon can participate in the myopathy of patients with DM through the STAT1 signaling pathway, resulting in muscle tissue damage [43]. Previous studies have confirmed that STAT1 is significantly overexpressed in the biopsy of patients with DM [42].

OAS1 belongs to the OAS gene family. OAS1 can regulate the expression of chemokines and IFN-responsive genes. In addition, studies have confirmed that OAS1 is a negative regulator of type I interferon signaling genes, which reminds us that OAS1 is closely correlated with type I interferon signaling pathway [44]. Related studies have shown that all OAS genes are regulated in patients with DM. In addition, 99% of OAS gene family networks are significantly upregulated [45]. IFI6 is an interferon stimulus that can be upregulated by the type I interferon gene. And with the research of IFI6 in recent years, it is widely accepted that IFI6 may play a critical role in regulating apoptosis and antiviral activity. Also, previous studies have suggested that in the muscle tissue of patients with DM, the mRNA expression level of IFI6 is significantly increased [9]. We notice that IFIT6 is involved in the IFN-*α*/*β* signaling pathway, which may have a close relationship with the regulation of viral infection in the pathogenesis of DM.

HLA-A is one of the sites on the HLA gene. Studies have shown that the strong expression of HLA-A in DM patients causes muscle fibers to be damaged by inflammatory cell infiltration, causing muscle damage [46]. At the same time, HLA-A participates in interferon-gamma signaling to modulate the immune response. Therefore, HLA-A is closely related to the inflammatory response of DM. TRIM25 belongs to the TRIM family and plays an important role in natural immune regulation. Studies have found that TRIM25 is significantly upregulated in the muscle tissue of patients with DM [47]. In addition, TRIM25 is closely associated with RIG-I, leading to the activation of RIG-I and stimulating the production of the antiviral cytokines IFN-*α* and IFN-*β*, which mediated signaling in the IFN pathways [48]. GBP1 is a kind of monomer engine protein composed of 593 amino acids. It is not expressed in normal human muscle tissue, so it may be a potential early warning signal for the occurrence and development of DM [49, 50].

Meanwhile, we conducted the interrelation analysis for the above vital hub genes that may be most closely associated with DM to elucidate their interactions [51–53]. As shown in Figure 6, the identified hub genes directly or indirectly communicated with each other via molecular signaling pathways related to response to type I interferon, response to virus, cellular response to interferon-gamma, and regulation of type I interferon production. ISG15, STAT1, IFIT3, IFI6, and OAS1 are mainly directly involved in and affect the type I interferon signaling pathway and response to virus, whereas HLA-A and DDX58 are mainly involved in the regulation of molecular mediator of immune response and cytokine production involved in immune response [54, 55]. ISG15, STAT1, TRIM25, and DDX58 are highly correlated with the positive regulation of type I interferon production. Moreover, ISG15, STAT1, and DDX58 are directly associated with the regulation of the ISGylation pathway in antiviral immunity [38]. In general, these findings suggested that these hub genes involved in the development of DM could be crucial factors in regulating the type I interferon system and the immune system. They could be new potential biomarkers and therapeutic targets.

In the present study, almost all the enrichment terms of upregulated genes in the GO enrichment analysis were related to chemical stimulus involved in sensory perception of smell. The KEGG pathway sensory perception of smell, enriched by upregulated genes, was consistent with the hypothesis that sensory perception of smell may participate in the regulation of DM. But there is not much literature involved. However, all the enrichment terms of downregulated genes in the GO enrichment analysis were associated with type I interferon and response to virus. The characteristics of patients with DM are mainly skin lesions and progressive muscle weakness [26], which are autoimmune diseases and are closely related to the enrichment of related type I interferon and viral response in GO downregulation enrichment analysis. Guanylate-binding protein 2 (GBP2) is a protein induced by IFN. It can mediate cellular immunity, participate in many biological responses, and increase IL-6 and IL-12. The secretion of TNF-*α* and other inflammatory factors plays an important role in the pathogenesis of DM [56]. Oligoadenylate synthase 3 (OAS3) is an essential antiviral protein, which can be induced by IFN and plays a key role in regulating protein synthesis and immune response [57]. Type I interferon is also involved in myopathy in patients with DM through the STAT1 signaling pathway. IFN-*α* and IFN-*β* can promote the expression of type I interferon-induced genes by combining IFNAR1 and IFNAR2 to activate the STAT1 signal transduction pathway, leading to muscle tissue damage [58, 59]. Therefore, from the analysis of GO downregulation enrichment, the above enrichment is significantly related to immune response.

What is more, the KEGG pathway-related virus infection was enriched by downregulated genes. It was consistent with the hypothesis that virus infection may participate in the regulation of DM. Therefore, viral infection and the occurrence and development of autoimmune diseases are of great significance in the pathogenesis of DM.

Last but not least, this study had several limitations. First of all, the etiology of this disease has not yet been fully clarified. It may be related to natural immunity, genetics, and viral infections. We need further data mining and bioinformatics analysis. In addition, the occurrence of clinical epithelial myositis is also affected by other factors, which will affect the specificity of the patient samples used for the study of DM. Moreover, further in vitro and functional studies and studies using knockout mice are needed to verify the molecular biological mechanisms of these genes.

## 5. Conclusions

Under the guidance of bioinformatics analysis, our research was aimed to identifying hub genes and molecular pathways involved in DM and pinpointing potential diagnostic or therapeutic biomarkers. In general, the present study identified 10 immune-related hub genes that were highly associated with the pathogenesis of DM, and it proposed that response to type I interferon, regulation of type I interferon production, and other immune response regulation pathways may be potential molecular pathways regulating DM. DM is mainly closely associated with the expression of proinflammatory factors and interferon family proteins and genes. Related inflammatory factors and interferon family proteins and genes participate in and mediate a variety of immune response pathways, which are of great significance for the in-depth study of the pathogenesis of DM and the clinical prevention and treatment of DM. In addition, further studies are needed to verify the associated hub genes and their functional roles in DM.

## Figures and Tables

**Table 1:** Top five Gene Ontology (GO) analysis terms for dermatomyositis (DM) enriched by upregulated and downregulated genes, respectively.

DEGs	ID	Term	Count	*P* value	Genes
Upregulated	GO:0050911	Detection of chemical stimulus involved in sensory perception of smell	23	3.79*E* − 22	OR10H5/OR10P1/OR1D2/OR1E2/OR1J4/OR2AT4/OR2B11/OR2L8/OR2T3/OR2T7/OR4C45/OR4K2/OR4X2/OR5D14/OR5I1/OR5R1/OR6K2/OR6N2/OR6Y1/OR8B2/OR8B3/OR8H2/OR8H3
	GO:0007608	Sensory perception of smell	23	1.79*E* − 21	OR10H5/OR10P1/OR1D2/OR1E2/OR1J4/OR2AT4/OR2B11/OR2L8/OR2T3/OR2T7/OR4C45/OR4K2/OR4X2/OR5D14/OR5I1/OR5R1/OR6K2/OR6N2/OR6Y1/OR8B2/OR8B3/OR8H2/OR8H3
	GO:0050907	Detection of chemical stimulus involved in sensory perception	23	6.48*E* − 21	OR10H5/OR10P1/OR1D2/OR1E2/OR1J4/OR2AT4/OR2B11/OR2L8/OR2T3/OR2T7/OR4C45/OR4K2/OR4X2/OR5D14/OR5I1/OR5R1/OR6K2/OR6N2/OR6Y1/OR8B2/OR8B3/OR8H2/OR8H3
	GO:0009593	Detection of chemical stimulus	23	3.79*E* − 20	OR10H5/OR10P1/OR1D2/OR1E2/OR1J4/OR2AT4/OR2B11/OR2L8/OR2T3/OR2T7/OR4C45/OR4K2/OR4X2/OR5D14/OR5I1/OR5R1/OR6K2/OR6N2/OR6Y1/OR8B2/OR8B3/OR8H2/OR8H3
	GO:0007606	Sensory perception of chemical stimulus	23	7.51*E* − 20	OR10H5/OR10P1/OR1D2/OR1E2/OR1J4/OR2AT4/OR2B11/OR2L8/OR2T3/OR2T7/OR4C45/OR4K2/OR4X2/OR5D14/OR5I1/OR5R1/OR6K2/OR6N2/OR6Y1/OR8B2/OR8B3/OR8H2/OR8H3
Downregulated	GO:0051607	Defense response to virus	48	8.69*E* − 22	ADAR/APOBEC3C/BST2/C1QBP/CXCL10/CXCL9/DDX58/DDX60/DNAJC3/DTX3L/EIF2AK2/FGL2/GBP1/HERC5/HTRA1/IFI16/IFI27/IFI44L/IFI6/IFIH1/IFIT1/IFIT2/IFIT3/IFIT5/IFITM1/ILF3/IRF9/ISG15/ITCH/LSM14A/MX1/MX2/OAS1/OAS2/OAS3/PARP9/PLSCR1/PTPRC/PYCARD/RSAD2/SAMHD1/SERINC3/STAT1/STAT2/TRIM22/TRIM25/TRIM38/ZC3HAV1
	GO:0009615	Response to virus	52	5.50*E* − 19	ADAR/APOBEC3C/BST2/C1QBP/CXCL10/CXCL9/DDX58/DDX60/DNAJC3/DTX3L/EIF2AK2/FGL2/FMR1/GBP1/HERC5/HSPB1/HTRA1/IFI16/IFI27/IFI44/IFI44L/IFI6/IFIH1/IFIT1/IFIT2/IFIT3/IFIT5/IFITM1/ILF3/IRF9/ISG15/ITCH/LSM14A/MX1/MX2/NPC2/OAS1/OAS2/OAS3/PARP9/PLSCR1/PTPRC/PYCARD/RSAD2/SAMHD1/SERINC3/STAT1/STAT2/TRIM22/TRIM25/TRIM38/ZC3HAV1
	GO:0060337	Type I interferon signaling pathway	29	9.03*E* − 19	ADAR/BST2/GBP2/HLA-A/HLA-E/IFI27/IFI6/IFIT1/IFIT2/IFIT3/IFITM1/IRF9/ISG15/LSM14A/MX1/MX2/OAS1/OAS2/OAS3/PTPN1/PTPN11/RSAD2/SAMHD1/SP100/STAT1/STAT2/UBE2K/USP18/XAF1
	GO:0071357	Cellular response to type I interferon	29	9.03*E* − 19	ADAR/BST2/GBP2/HLA-A/HLA-E/IFI27/IFI6/IFIT1/IFIT2/IFIT3/IFITM1/IRF9/ISG15/LSM14A/MX1/MX2/OAS1/OAS2/OAS3/PTPN1/PTPN11/RSAD2/SAMHD1/SP100/STAT1/STAT2/UBE2K/USP18/XAF1
	GO:0034340	Response to type I interferon	29	2.44*E* − 18	ADAR/BST2/GBP2/HLA-A/HLA-E/IFI27/IFI6/IFIT1/IFIT2/IFIT3/IFITM1/IRF9/ISG15/LSM14A/MX1/MX2/OAS1/OAS2/OAS3/PTPN1/PTPN11/RSAD2/SAMHD1/SP100/STAT1/STAT2/UBE2K/USP18/XAF1

GO: Gene Ontology.

**Table 2:** The top five Kyoto Encyclopedia of Genes and Genomes (KEGG) pathways enriched in dermatomyositis (DM) by upregulated and downregulated genes, respectively.

DEGs	ID	Pathway	Count	*P* value	Genes
Upregulated	hsa04740	Olfactory transduction	23	1.10*E* − 24	OR10H5/OR10P1/OR1D2/OR1E2/OR1J4/OR2AT4/OR2B11/OR2L8/OR2T3/OR2T7/OR4C45/OR4K2/OR4X2/OR5D14/OR5I1/OR5R1/OR6K2/OR6N2/OR6Y1/OR8B2/OR8B3/OR8H2/OR8H3
Downregulated	hsa05164	Influenza A	27	1.07*E* − 08	ACTG1/ADAR/CCL2/CXCL10/DDX58/DNAJC3/EIF2AK2/EP300/HLA-DRA/IFIH1/IL18/IRF9/JAK2/MAPK1/MX1/MX2/OAS1/OAS2/OAS3/PIK3R1/PYCARD/RAB11A/RSAD2/STAT1/STAT2/TNFSF10/TRIM25
	hsa05012	Parkinson disease	31	5.57*E* − 08	ATP5F1C/ATP5PD/CALML3/CALML5/COX5A/COX7B/COX7C/DUSP1/GNAI3/HSPA5/KIF5B/KLC1/NDUFA1/PSMA3/PSMA6/PSMA7/PSMB1/PSMB4/PSMC5/PSMD1/PSMD12/PSMD7/TUBB2A/TXN/UBA1/UBA52/UBB/UBE2J1/UBE2L6/UQCR10/UQCRC2
	hsa05167	Kaposi sarcoma-associated herpesvirus infection	27	1.53*E* − 07	CALML3/CALML5/CLEC2B/EIF2AK2/EP300/GABARAPL2/GNB4/GNG11/GNG12/GSK3B/HIF1A/HLA-A/HLA-E/IL6ST/IRF9/JAK2/MAPK1/NRAS/PIK3R1/RAC1/RB1/STAT1/STAT2/STAT3/UBA52/UBB/ZFP36
	hsa05169	Epstein-Barr virus infection	27	4.41*E* − 07	B2M/CALR/CD44/CXCL10/DDX58/EIF2AK2/HLA-A/HLA-DRA/HLA-E/IRF9/ISG15/MDM2/OAS1/OAS2/OAS3/PIK3R1/PSMC5/PSMD1/PSMD12/PSMD7/RAC1/RB1/RIPK1/STAT1/STAT2/STAT3/USP7
	hsa04144	Endocytosis	30	8.48*E* − 07	ARF4/ARPC1A/ARPC2/ARPC5/CHMP1B/CHMP4B/CHMP5/CLTC/DAB2/HLA-A/HLA-E/HSPA8/ITCH/KIF5B/MDM2/RAB10/RAB11A/RAB31/RAB7A/RAB8A/RHOA/SH3GLB1/SMURF2/SNX6/TSG101/VPS29/VPS35/VTA1/WASHC4/WASHC5

KEGG: Kyoto Encyclopedia of Genes and Genomes; DEGs: differentially expressed genes.

**Table 3:** Hub genes of the top two subclusters identified for dermatomyositis (DM).

Gene	Degree	DEGs	LogFC	Subcluster
CXCL10	66	Down	-5.42	Cluster 1
DDX58	67	Down	-3.34	Cluster 1
GBP1	59	Down	-3.01	Cluster 1
IFIT3	59	Down	-4.20	Cluster 1
ISG15	89	Down	-4.04	Cluster 1
IFI6	46	Down	-3.81	Cluster 1
OAS1	60	Down	-3.49	Cluster 1
STAT1	98	Down	-3.87	Cluster 1
TRIM25	50	Down	-3.08	Cluster 2
HLA-A	47	Down	-2.58	Cluster 2

LogFC: log fold change.

## Data Availability

All data were obtained from the Gene Expression Omnibus (GEO) database.

## Supplementary Materials

Supplementary Materials Supplementary Material 1: all upregulated genes and downregulated genes are filtered out by running the limma package in R.
