# Supplementary material for: Identification of Vital Hub Genes and Potential Molecular Pathways of Dermatomyositis by Bioinformatics Analysis
Source: Biomed Res Int. 2021 Sep 18;2021:9991726. doi: 10.1155/2021/9991726 (PMC8464434; doi:10.1155/2021/9991726)
Supplement: Supplementary Materials — Supplementary Material 1: all upregulated genes and downregulated genes are filtered out by running the limma package in R. [file 9991726.f1.docx]

**Table1.** The 915 DEGs filtered out from Gene expression profile data, including 167 upregulated genes and 748 downregulated genes compared with DM and the health controls.

| DEGs | Gene names |
| --- | --- |
| Upregulated DEGs | MIR4262, MIR3689E, MIR4418, MIR4325, MIR3689D1, MIR3689F, FAM197Y9, MIR4681, MIR548I2, LINC01377, OR8H2, MIR544A, LOC100507412, MUCL3, MIR4301, SMR3A, FAM242F, FAM197Y7, MIR890, SNORA25B, PRR20D, MIR3194, LOC642929, LOC105374297, ROCK1P1, RNU6-8, OR8B2, LOC440896, SNORA75B, FAM106A, FCGR1B, USP41, PRR20A, PRR20C, MIR587, REXO1, MIR1269A, MIR4704, RHPN2, DAZ4, ACR, SNORD65C, C4A-AS1, MIR4789, FAM197Y6, LOC101928739, MIR4692, SNORD114-30, MIR20B, LINC00434, MIR3670-1, MIR3670-2, SNORD82, EWSAT1, CDRT7, MIR548H1, MIR577, MIR3148, DAZ2, RPL23P8, BCRP3, ZNF733P, OR4C45, LOC105372582, MIR4518, CTIF, FRG2DP, LOC440040, OR10H5, MIR4514, OR2AT4, MIR646, LINC02495, RNASE3, RNASE8, OR2L8, LINC01628, OR1E2, LOC728743, MIR2114, LINC01040, LOC100286906, RBMY1D, TP53TG3C, MIR204, MIR3666, MIR548M, LINC00619, TTTY8, TTTY8B, HAO2-IT1, TEKT4P2, MIR3659, SLC35G6, RBMY1E, FLJ43315, MIR4438, FCRL5, MIR4300, PMCHL1, MIR892C, MUC3A, TSPY10, LOC100506274, BAGE2, MIR7-3, LINC01656, LINC00508, FRG1-DT, TP53TG3, PF4, LINC02280, OR6Y1, LOC285889, SLC35G3, OR10P1, MUC22, MKRN9P, PSG6, LINC01151, OR5I1, OR2T7, CGB7, LINC00440, OR5D14, OR2B11, OR4K2, LOC102467217, OR2T3, NAALADL2-AS1, ECRP, MIR3150A, MIR3161, HYDIN2, RNU6-56P, MIR626, OR5R1, SNORD7, MYRFL, SNORD74B, CXADRP2, ZNF813, SNORD28B, PLEKHA8P1, HMGB3P1, OR6N2, ANP32D, LOC442028, PRG1, SNORD114-18, LINC00376, KRTAP15-1, MIR4311, LINC01766, OR8B3, OR6K2, SCGB1C1, FAM106B, OR1D2, RNVU1-20, SPRY4-AS1, OR4X2, OR1J4, OR8H3, MIR338, MIR4442, LOC100506384 |
| Down-regulated DEGs | CXCL10, S100A9, LCE3D, SPRR2G, LCE2C, CXCL9, SERPINB3, IFI44, LCE3E, IFI44L, VTRNA1-1, PARP14, IFIT3, S100A7, IFIH1, ISG15, IVL, S100A8, IFIT1, STAT1, RHOQ, IFI6, SPRR1B, SPRR1A, LCE2D, SNORA24, CCL2, DSG3, FGL2, HLA-DRA, CALR, XIST, PSAP, PI3, MX1, SNORA5A, KRT16, TMSB10, OAS1, GBP4, HSP90B1, SNORA22, EIF2AK2, RPL27, B2M, DDX58, SCARNA17, SNORD116-3, TXN, ZFP36, LCE6A, YWHAZ, SLPI, LCE1F, BST2, RAB8B, SERPINB5, NMI, LUM, CSTA, OAS2, TSIX, SNORA50C, TAF1D, LINC00302, TRIM25, NOP10, ROCK1, DSC1, TAF13, CYBB, ITM2B, LYZ, OAZ1, LCE2A, CLCN3, SAMD9L, EIF4H, WFDC12, ABCA12, SPINK5, LCE1C, DNAJA1, SNHG14, SNORD116-9, CHMP5, GBP1, NDEL1, PYURF, HERC5, SNORD116-17, RAB8A, SAT1, LCE1B, LCE2B, IFITM1, SNORD104, TRA, DSG1, TAOK1, OAS3, MSN, IFI16, GABARAPL2, SAMHD1, DSC3, PARP4, PLSCR1, GBP5, CDSN, SNORA68, TNFSF10, IFIT2, SERPINB12, PCBP1, GJA1, INTS5, SLURP1, LCE1A, PSMD7, CLCA2, PARP12, ARRDC3, KPRP, SNORD36B, SNORD3B-1, SENP6, KIF5B, RAB11A, TMED2, LLPH, PSMA3, CD24, ELOVL4, SNORA79, ELF1, PARP9, UQCC2, CD44, RNF213, HSBP1, ARG1, ELOC, SNORD47, SNORD56B, SMCHD1, USP18, CYB5A, UQCR10, UBE2K, VPS35, SNORD36A, PTPRC, EDF1, EPSTI1, IL6ST, CXCL11, HEXB, MIR3671, ATP5PD, PCNA, CAPNS1, GBP1P1, LAPTM5, SNORD41, MMP24OS, LINC01527, SNORA4, SH3GLB1, SNORA14A, RHOA, TUBB2A, HSPA5, HAL, CTSS, LOC220729, FAM160A1, CRCT1, GNG12, CRYBG1, SHOC2, OSTC, MRPL35, RNASE7, GLO1, OXLD1, CYLD, COX7B, PDCD5, ANKRD10, EIF3A, NPC2, CDR1, MYSM1, SERPINB4, HTRA1, SQOR, RSAD2, AHR, PTBP3, CHMP1B, HLA-A, MIR421, SNORD28, CSNK2A2, C6orf62, SBDS, LAP3, DTX3L, IL18, POLR2I, TUT7, POF1B, MAT2A, IFI27, MASTL, ANXA1, CLINT1, C19orf53, SNORA22C, SNORA28, RAB31, RAB12, GSK3B, PPP2R5C, TAX1BP1, GALNT1, STAT2, MDM2, CLEC2B, ITGA6, SNORD55, ZRANB2, MBNL2, ACTR3, EIF4EBP2, ABI1, PEA15, ETV6, SNORA37, SNHG16, HSPB1, JUNB, SBSN, FNBP1, AKIRIN2, SERPINF1, HIF1A-AS2, COL6A3, PALMD, DKFZP586I1420, IFI30, GPR87, ZFP36L2, FAM83B, RBBP8, RHOT1, CD164, CTSK, SP100, PPP4R3A, FN1, SYNGR2, MMP2, TCEAL9, PIK3R1, ZBED6, SNORD116-19, MIR1184-1, MIR1184-2, MIR1184-3, ACTG1, C1orf43, PYCARD, VAPA, LEO1, FBN1, ZNF770, DSC2, WASHC4, KRT80, MRPS21, CASP14, LINC01881, TPM4, MIR3064, XAF1, XRN1, ARPP19, MXRA5, FLG, RANBP9, FGF7, FCER1G, CHIC2, DMKN, BPTF, TIMM23, SNRPD1, SPRR2B, WASF2, FTL, GOLIM4, APOBEC3C, ATP6V0E1, C1QA, DDX60L, DNAJC7, TRIR, TM9SF2, CD163, LAMP2, SERINC3, FPR3, GOLGA5, PELI1, KDM7A, SNORA12, MIR5047, RPN2, SLC39A6, SRP72, BLMH, HECTD1, CLTC, RAB11B-AS1, ZNF430, BLOC1S2, PLS3, DDX60, SCARB2, AZGP1, ANXA2P2, HNRNPDL, GRN, SLK, FNBP4, LGALS3BP, TYMS, SFRP2, RUFY3, SNORA71D, XRCC5, EIF2A, DYNLT1, MUC4, UBE2B, UBE2J1, PSMB1, EZR, SUGT1, LGMN, SF3B1, ELL2, ALOX12B, UBE2L6, SMG1P3, HOOK3, MT2A, SNORA49, MAIP1, SERPINB13, PSMA7, UCHL3, PSMD1, SNHG8, RAC1, CAST, CDC5L, UBE2R2, PPL, OGT, RNF11, C1orf52, HNRNPU, CALML5, PSMD12, DIS3, SMC5, CHMP4B, PLBD1, SCARNA3, POMP, SNORD87, TOP2B, CDH1, BAZ1A, HIF1A, N4BP2L2, XPO6, CCDC47, LIN7C, ATP5F1C, TYROBP, GPR137B, SOD1, SMIM26, SNORD80, SNORA41, GDI2, ZNF750, GNAI3, YBX1, JAK2, LGALSL, FLG2, GAS5, CD59, SNORA63D, BCAS2, CXCL14, FOPNL, ILF3, IRF9, HSPA8, KRIT1, RAD50, USP7, HADHA, DDX6, DIAPH2, SAP18, ARPC1A, TXNIP, SPTBN1, SPRR2E, C1QBP, EIF4G2, GTF2I, ARGLU1, NRIP1, PTPN12, SERPINB2, KLK7, PKM, APOD, HERC6, GID8, COPB2, MIR590, PITPNB, PTPN13, CBR3, CMPK2, GTF2IP1, IER3IP1, THOC7, MORF4L2, KRT23, OGFRL1, NT5E, MGC4859, UGCG, UBALD2, CRABP2, APLP2, C19orf33, SNORD116-1, PTBP1, ACSL4, CSDE1, MACF1, SF3B6, TRIM22, ZNFX1, STAU1, BMPR2, IDE, MYO1D, DUSP1, DPY30, APPL2, ZC3HAV1, MBD2, CHD1, DCN, SAMD9, EXT1, CTSA, MMADHC, LONP2, GPBP1, FAT1, UBA2, CUL1, SORD2P, KRTDAP, BTN3A3, AFTPH, AKAP8L, EFCAB14, NRDC, PRDX6, WIPI1, GLTP, CD74, CCNL1, NAA50, GTF2H2C_2, SNX6, MAFB, XG, PEX3, NCKAP1, SNORD114-1, CPM, MS4A7, SFPQ, COX5A, VAMP3, SUPT16H, UBA1, SYF2, RPF1, RAB7A, IFIT5, ERBIN, HNRNPM, PLPP1, API5, SPARCL1, CD47, HIGD1A, WSB1, IQGAP1, DSP, KLC1, UBL3, VPS29, MED13, ITCH, MYH9, NFIB, COX7C, CPA4, WASHC5, TMEM45A, TRA2B, PTPN1, ARNTL2, LIPK, TIMM13, UQCRC2, SNORD110, CHD2, TOMM6, LAMP1, IPO7, NUFIP2, SNORA64, PRRC2C, MTCH2, TMEM126A, RAB27B, NUCKS1, SP110, MIR553, MT1F, LSM14A, NFAT5, CAP1, DRG1, EIF1, SPRED1, SNORA26, SPG11, LMAN1, PNISR, BASP1, GBP2, STAT3, SFN, EIF2S2, EFNB2, DNAJC2, SMC3, DAB2, PICALM, ARCN1, SNORD44, SRSF10, DNAJC3, NTAN1, GOLGA4, MBNL1, GNG11, GJB2, VAMP8, SNORD99, ADAP2, EIF3J, DEK, MAF, SNORD2, EFEMP1, CDC73, NDUFA1, ANXA5, FMR1, STX12, MGME1, KLHDC7B, ECM2, ARHGAP21, EP300, IFFO2, RIPK2, ATP6V1C2, SCEL, ZNF791, SNX9, SNAI2, CSNK1G2, HDDC2, DYNC1H1, HBA2, ZNF117, LUC7L3, TNC, CDC42BPB, MAPK1, UTRN, DDX42, STRN3, MXD1, RPS6KA3, THBS1, TRIP11, AFF4, CAPNS2, CFH, PTRH2, MFSD14B, DHX29, PSMB4, ESRP1, EMP3, ELOB, ASPN, ANGPTL1, ARL6IP5, NFE2L3, KDELR2, TMEM30A, LUC7L, UBE2D3, RB1, COPA, GOLGA8A, CENPC, HSBP1L1, POSTN, MNDA, CD68, SMURF2, RAB10, ARPC2, TOP1, TRIM38, KHDRBS1, ARPC5, SERPING1, NSA2, EPPK1, APCDD1, PRPF4B, DAAM1, HLA-E, PPA1, MIR1282, VTA1, SCARNA10, TAF7, SKP1P2, CXCR2P1, WDR45B, TNKS2, GNB4, APPL1, AKAP9, UBA52, ADAR, ARF4, C16orf70, MPHOSPH6, RIPK1, DNAJC15, PANK3, CSE1L, TRIP12, RBMS1, SPECC1L, LCP2, CRLF3, TRAPPC8, HSPA9, SNORA48, CNFN, TPR, HMGCR, SDAD1, MIR3661, CXCL16, SCP2, FBXO11, MX2, THRAP3, MARK3, CCNI, MYCBP2, PSMC5, NCOA4, MCUR1, TMEM33, PTGES3, MAN1A2, BUB3, PTPN11, DEGS1, CNOT1, NEAT1, PSMA6, MACO1, GGCT, SF3B3, DOCK11, STOM, RPL18A, SNORD105B, MIF, TULP4, BOD1L1, SEC11C, SUCO, FKBP3, UBB, SYNE2, ASDURF, PHACTR4, NAPA, TWF1, TSG101, SPTY2D1, DDX24, NBR2, CALML3, DHX15, NRAS |

According to the absolute value of the logarithmic change(logFC), the up-regulated and down-regulated genes are arranged in descending order. DEGs – differentially expressed genes; FC – fold change.
